# Lymphocytic thrombophilic arteritis with lower-limb ulcers^☆^^☆☆^

**DOI:** 10.1016/j.abd.2020.08.012

**Published:** 2021-03-15

**Authors:** Jessica Shen Tsy Wu Kim, Valeria Romero Godofredo, Milvia Maria Simões e Silva Enokihara, Mônica Ribeiro de Azevedo Vasconcellos

**Affiliations:** a Department of Dermatology, Universidade Federal de São Paulo, São Paulo, SP, Brazil; b Department of Pathology, Universidade Federal de São Paulo, São Paulo, SP, Brazil

**Keywords:** Arteritis, Skin ulcer, Vasculitis

## Abstract

Lymphocytic thrombophilic arteritis is a recently described entity, histopathologically characterized by lymphocytic vasculitis that affects the arterioles of the dermo-hypodermic junction, associated with deposition of fibrin and a luminal fibrin ring. A 49-year-old female patient presented with achromic maculae and a well-defined ulcer on the medial aspect of the left lower limb. The biopsy showed intense inflammatory infiltrate in the papillary dermis with a predominance of lymphocytes, and medium-caliber vessels surrounded by mononuclear infiltrates in the deep reticular dermis. Masson’s trichrome staining showed intense destruction of the muscle layer of the vascular wall and a fibrin ring. Good clinical response was attained with azathioprine. The authors believe that the ulceration might be another clinical presentation or represent an atypical progression of this condition.

## Introduction

Lymphocytic thrombophilic arteritis (LTA), or macular lymphocytic arteritis, is a recently described entity, histopathologically characterized by lymphocytic vasculitis that affects arterioles of the dermo-hypodermic junction, associated with the deposition of fibrin and a luminal fibrin ring.^1^, ^2^ We describe a case of LTA with ulcers in the lower limbs.

## Case report

A previously healthy 49-year-old female patient started to show erythematous maculae on her lower limbs 17 months ago and had been followed at our service for 11 months. She had been treated at another service with prednisone 0.5 mg/kg//day for a short period after a skin biopsy, which resulted in the development of an ulcer.

**Figure 1:**
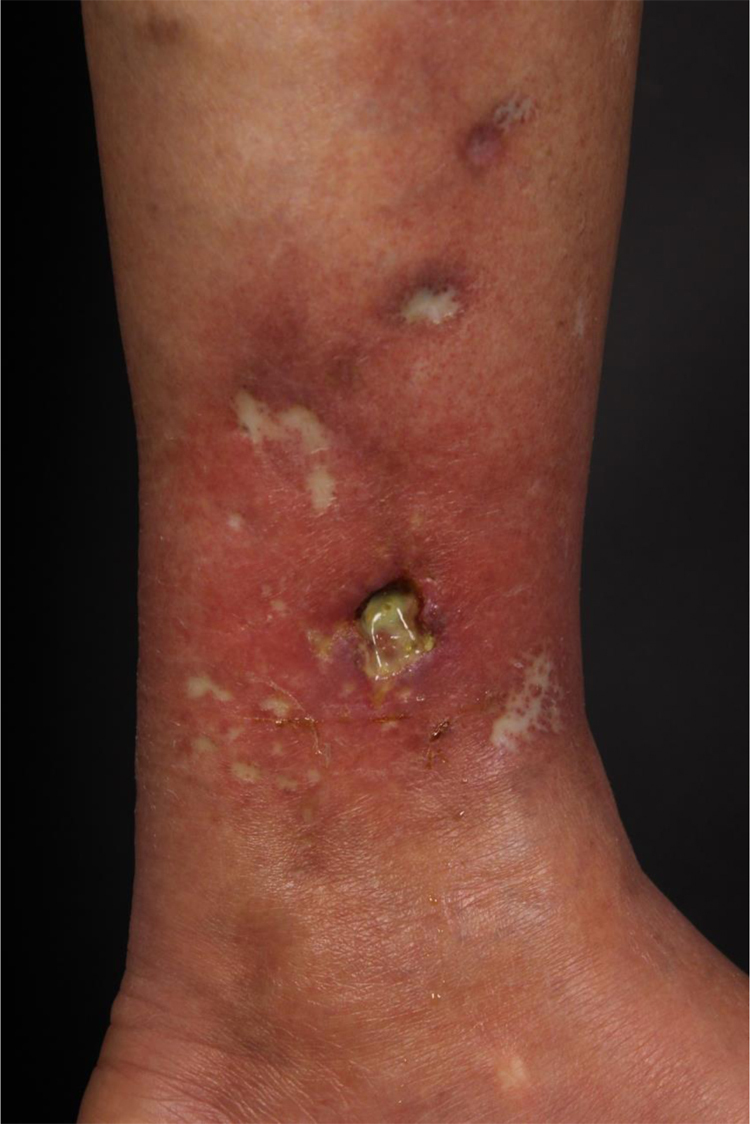
Achromic maculae with well-defined edges, Milian’s white atrophy, erythematous maculae, and an ulcer measuring 1.5 cm in diameter, on the medial aspect of the left lower limb.

At the examination, she had well-defined achromic maculae, compatible with Milian’s white atrophy and erythematous maculae, in addition to an ulcer measuring 1.5 cm in diameter, with well-defined and erythematous edges with a fibrinous bottom, on the medial aspect of the left lower limb (Fig. 1).

**Figure 2:**
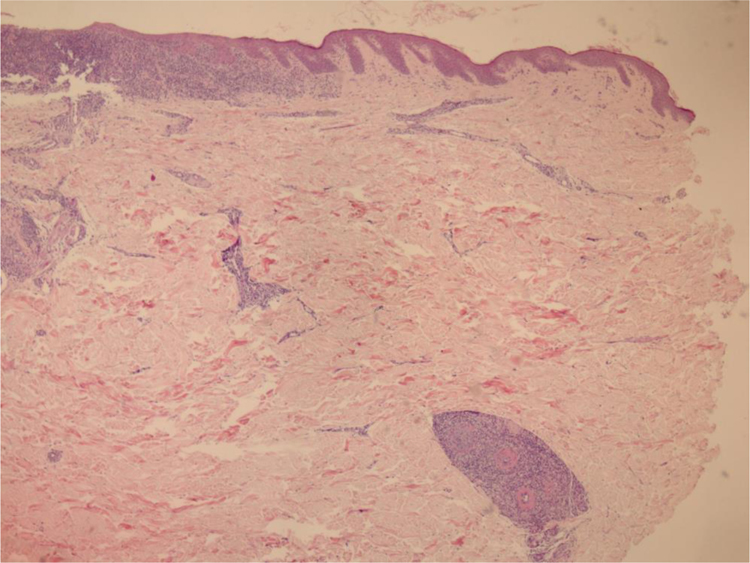
In the papillary dermis, intense inflammatory infiltrate with a predominance of lymphocytes is shown, without compromising the epidermis. In the deep reticular dermis, medium-caliber vessels surrounded by mononuclear infiltrates are shown (Hematoxylin & eosin, ×40).

**Figure 3:**
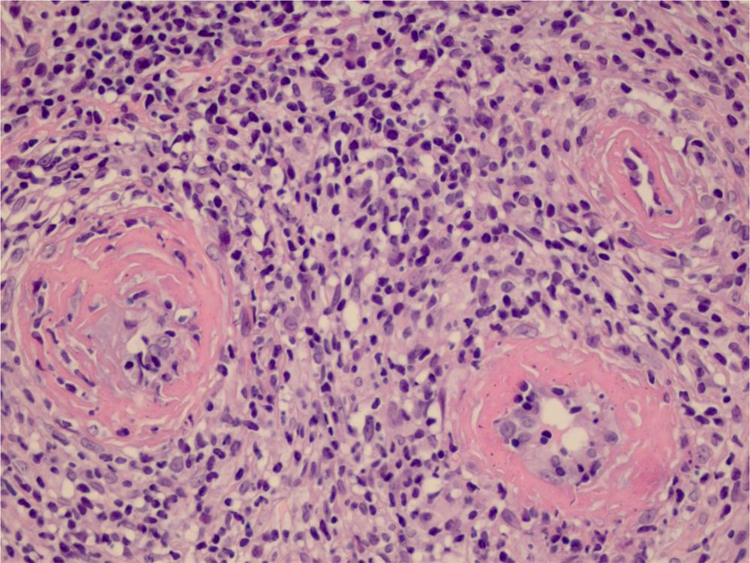
The detail shows three vessels with wall involvement by the inflammatory infiltrate, with a predominance of lymphocytes and an amorphous eosinophilic substance in the lumen, suggestive of fibrin (Hematoxylin & eosin, ×400).

**Figure 4:**
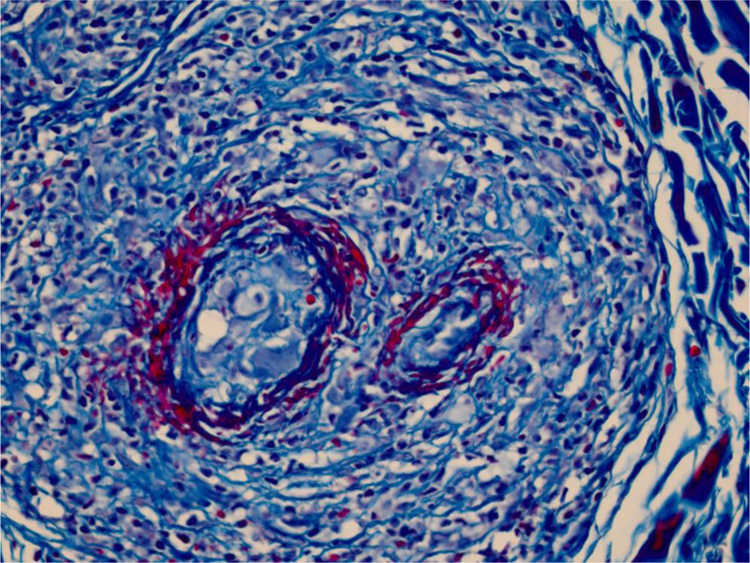
The use of Masson’s trichrome staining shows that the destruction of the vascular wall and muscle layer was intense, since no smooth muscle fiber stained in red is observed, and the amorphous material in the lumen is stained red, characterizing the aspect of “fibrin ring” (Masson’s trichrome, ×400).

**Figure 5:**
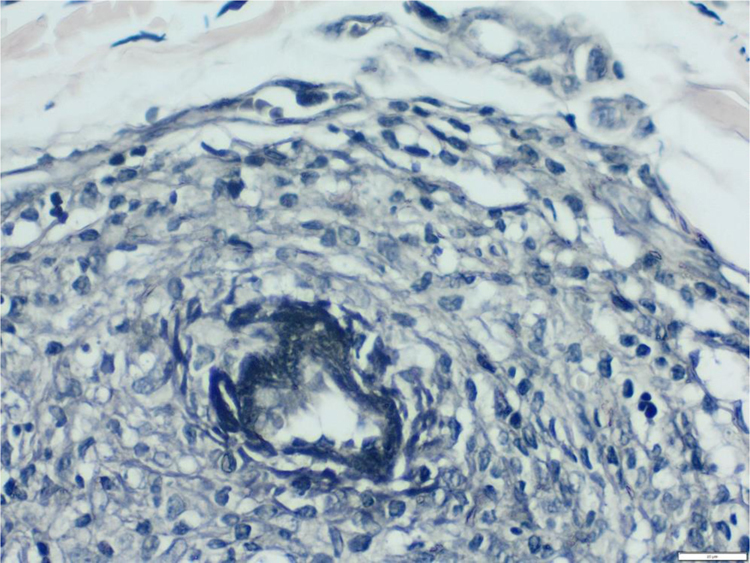
Using Verhoeff's method for elastic fibers, it can be observed that elastic fibers are found close to the lumen and interspersed with the “fibrin ring”, suggesting that these vessels are arteries and characterizing the histological picture of lymphocytic thrombophilic arteritis (Verhoeff, ×400).

We chose not to perform a new biopsy due to the patient’s poor circulation status. A review of the initial biopsy showed intense lymphocytic inflammatory infiltrate in the papillary dermis (Fig. 2), vessels with a predominantly lymphocytic inflammatory infiltrate in the deep reticular dermis and the presence of fibrin (Fig. 3) in the lumen. Masson’s trichrome staining showed the amorphous material stained in red, characterizing the aspect of a “fibrin ring” (Fig. 4). The Verhoeff staining method showed elastic fibers close to the lumen and interspersed in the “fibrin ring”, characterizing arteries, with a histopathological picture of LTA (Fig. 5).

Laboratory tests such as ANA, anti-DNA, anticardiolipin antibody, Antibodies to Extractable Nuclear Antigens (anti-ENA), Anti-Neutrophil Cytoplasmic Antibodies (ANCA), complement, cryoglobulins, Erythrocyte Sedimentation Rate (ESR), as well as general exams and liver and kidney function tests were normal. Arterial and venous Doppler ultrasound assessment disclosed venous insufficiency in the left lower limb.

**Figure 6:**
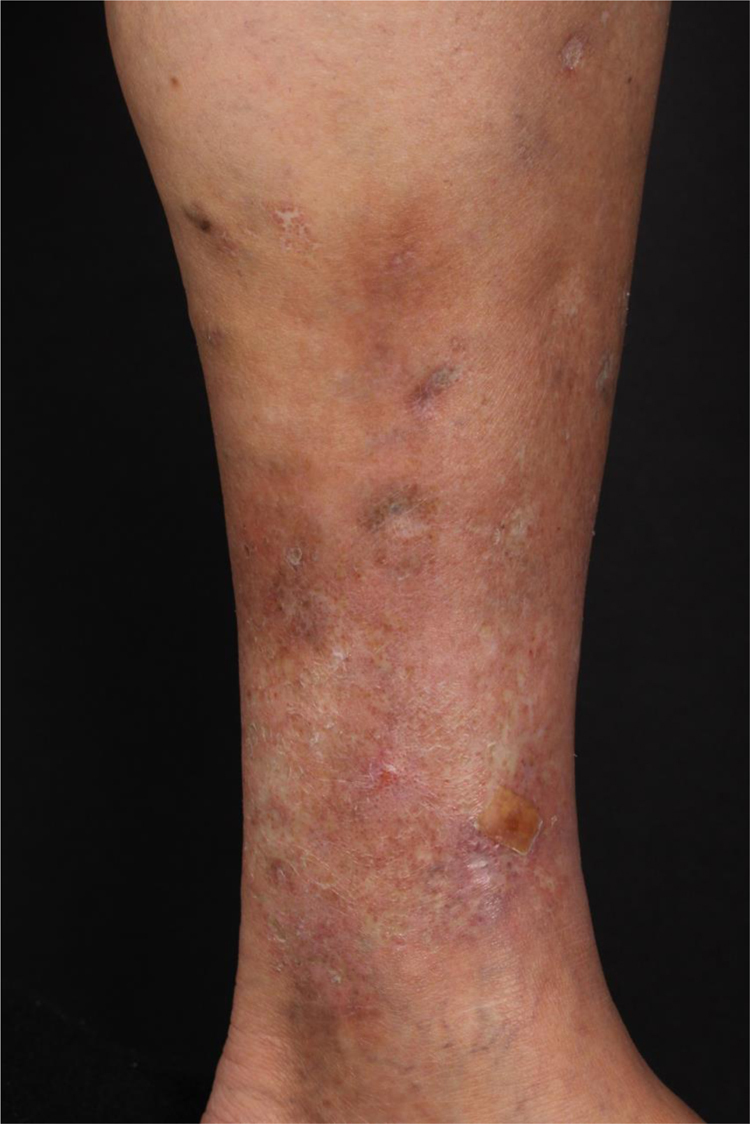
Good clinical response after increasing the azathioprine dose to 250 mg.

Three short cycles of prednisone (0.5 mg/kg/day) were administered and 50 mg/day of dapsone was started, gradually increasing the dose to 150 mg/day. However, the patient developed spontaneous ulcers in the lower limbs. Dapsone was discontinued and azathioprine 100 mg/day was introduced, in addition to 20 mg/day of prednisone and maintenance of local care, with ulcer healing and pain relief in one month. After 5 months of follow-up, new lesions appeared, and an increase in the dose of azathioprine to 250 mg was prescribed, finally showing a good clinical response (Fig. 6).

## Discussion

LTA was first described as a macular arteritis in 2003, by Fein et al., after the report of three female patients with an asymptomatic hyperpigmented macular eruption, with no consistent association with serological abnormalities or autoimmune diseases of the connective tissue.^3^ Histopathologically, it was characterized as a lymphocytic arteritis in the reticular dermis and hypodermis, with luminal occlusion.^3^

Subsequently, Lee et al. suggested the term LTA, due to the heterogeneity of clinical presentation with the same histopathological alterations. Its association with Cutaneous Polyarteritis Nodosa (cPAN) has been debated since the entity was described, and there is no consensus in the literature.^2^ Ishibashi and Chen described arteritis alterations in patients with cPAN as a four-stage process (acute, subacute, under repair, and healed). In the subacute stage, concentric fibrinoid necrosis of the intima and polymorphous inflammatory infiltrate comprising not only neutrophils, but also lymphocytes and histiocytes have been described.^4^ Classically, LTA is associated with a dense lymphocytic infiltrate and a luminal fibrin ring.^5^ Although there is no consensus in the literature, some authors believe that LTA is a latent form or a final stage of PAN, or even a different entity.^6^, ^7^ No association with this condition has been observed so far in our patient.

As for the clinical picture, hyperchromic macules in the lower limbs and, more rarely, in the upper limbs are observed, especially in women over 40 years old, in addition to livedo racemosa/reticularis or nodules, reinforcing the clinical similarity to PAN. The reported patient was in the age range compatible with the previously described cases, with maculae and ulcers, but without livedo or nodules. Moreover, the patient also had poor local circulation.^2^, ^3^, ^8^, ^9^ LTA can be associated with thrombophilic alterations, such as increased antiphospholipid antibodies and anticardiolipin antibodies, increased antinuclear antibodies, in addition to elevated ESR, findings not observed in the described case.^2^, ^8^

Due to the scarcity of cases in the literature, there is still no consensus regarding the therapeutic options for this new entity. There are reports of treatment with prednisone alone or in combination with cyclophosphamide, atovaquone, pentoxifylline, aspirin/doxycycline, dapsone, or narrow-band ultraviolet light B (NBUVB), in addition to reports of improvement in untreated lesions.^1^, ^5^, ^9^

After a literature review, two LTA reports were found, showing ulcers in the lower limbs, which were histopathologically compatible with the diagnosis.^9^, ^10^ Llamas-Velasco et al. described a case of a 25-year-old woman without venous insufficiency signs and symptoms, who developed a non-traumatic ulcer on the lower limbs with an LTA-compatible biopsy, which showed clinical improvement after 42 months of treatment with pentoxifylline and prednisone.^10^ The authors believe the ulcer was caused by ischemic damage, secondary to the presence of a chronic lymphocytic vascular lesion, associated with local conditions, such as poor collateral blood circulation or increased venous pressure.^10^ In the present case, the biopsy was performed on a macular lesion, which developed into an ulcer after the procedure and Doppler ultrasound assessment showed venous insufficiency, which could indicate an ulcerous lesion secondary to stasis; however, one can also speculate it was an atypical evolution of arteritis, as it was painful and healed with immunosuppressive therapy, in addition to the fact that venous insufficiency has already been implicated in the literature as a possible etiopathogenic factor of LTA.^10^

Clinically, differentiation from cPAN is also possible, where painful ulcerated lesions are more characteristic and their resolution depends on aggressive immunosuppressive treatment. In this case, the differentiation was histopathological, but one cannot rule out the association between the two conditions, previously mentioned as being possibly associated.

The present report describes a rare case of a middle-aged Caucasian woman with painful ulcerations, which may be another clinical presentation of LTA or represent an atypical progression of this condition. Moreover, this condition usually has an indolent course with variable response to treatment, sometimes showing improvement without any treatment, unlike the reported patient, who only responded after several months of 250 mg/day of azathioprine.

## Financial support

None declared.

## Authors’ contributions

Jessica Shen Tsy Wu Kim: Approval of the final version of the manuscript; design and planning of the study; drafting and editing of the manuscript; collection, analysis, and interpretation of data; critical review of the literature; critical review of the manuscript.

Valeria Romero Godofredo: Approval of the final version of the manuscript; design and planning of the study; drafting and editing of the manuscript; collection, analysis, and interpretation of data; critical review of the literature; critical review of the manuscript.

Milvia Maria Simões e Silva Enokihara: Approval of the final version of the manuscript; design and planning of the study; drafting and editing of the manuscript; collection, analysis, and interpretation of data; effective participation in research orientation; critical review of the literature; critical review of the manuscript.

Mônica Ribeiro de Azevedo Vasconcellos: Approval of the final version of the manuscript; design and planning of the study; drafting and editing of the manuscript; collection, analysis, and interpretation of data; effective participation in research orientation; intellectual participation in the propaedeutic and/ or therapeutic conduct of the studied cases; critical review of the literature; critical review of the manuscript.

## Conflicts of interest

None declared.
